# IDH-mutant glioma specific association of rs55705857 located at 8q24.21 involves MYC deregulation

**DOI:** 10.1038/srep27569

**Published:** 2016-06-10

**Authors:** Yavuz Oktay, Ege Ülgen, Özge Can, Cemaliye B. Akyerli, Şirin Yüksel, Yiğit Erdemgil, İ. Melis Durası, Octavian Ioan Henegariu, E. Paolo Nanni, Nathalie Selevsek, Jonas Grossmann, E. Zeynep Erson-Omay, Hanwen Bai, Manu Gupta, William Lee, Şevin Turcan, Aysel Özpınar, Jason T. Huse, M. Aydın Sav, Adrienne Flanagan, Murat Günel, O. Uğur Sezerman, M. Cengiz Yakıcıer, M. Necmettin Pamir, Koray Özduman

**Affiliations:** 1 Brain Tumor Research Group, Acibadem University, Istanbul, Turkey; 2 Department of Molecular Biology and Genetics, Faculty of Arts and Sciences, Acibadem University, Istanbul, Turkey; 3 Department of Medical Biology, School of Medicine, Acibadem University, Istanbul, Turkey; 4 Izmir International Biomedicine and Genome Institute (iBG-izmir), Dokuz Eylul University, Izmir, Turkey; 5 Faculty of Engineering, Department of Medical Engineering, Acibadem University, Istanbul, Turkey; 6 Department of Biological Sciences and Bioengineering, Faculty of Engineering and Natural Sciences, Sabanci University, Istanbul, Turkey; 7 Department of Neurosurgery, School of Medicine Yale University, New Haven, CT 06520, USA; 8 Functional Genomics Center Zurich, UZH/ETH, Zurich, Switzerland; 9 Cancer Institute, University College London, 72 Huntley Street, WC1E 6DD, London, UK; 10 Department of Radiation Oncology, Memorial Sloan Kettering Cancer Center, New York, NY 10065, USA; 11 Human Oncology and Pathogenesis Program, Memorial Sloan Kettering Cancer Center, New York, NY 10065, USA; 12 Department of Medical Biochemistry, School of Medicine, Acibadem University, Istanbul, Turkey; 13 Department of Pathology, Memorial Sloan Kettering Cancer Center, New York, NY 10065, USA; 14 Department of Pathology, School of Medicine, Acibadem University, Istanbul, Turkey; 15 Department of Medical Statistics and Bioinformatics, School of Medicine, Acibadem University, Istanbul, Turkey; 16 Department of Neurosurgery, School of Medicine, Acibadem University, Istanbul, Turkey

## Abstract

The single nucleotide polymorphism rs55705857, located in a non-coding but evolutionarily conserved region at 8q24.21, is strongly associated with IDH-mutant glioma development and was suggested to be a causal variant. However, the molecular mechanism underlying this association has remained unknown. With a case control study in 285 gliomas, 316 healthy controls, 380 systemic cancers, 31 other CNS-tumors, and 120 IDH-mutant cartilaginous tumors, we identified that the association was specific to IDH-mutant gliomas. Odds-ratios were 9.25 (5.17–16.52; 95% CI) for IDH-mutated gliomas and 12.85 (5.94–27.83; 95% CI) for IDH-mutated, 1p/19q co-deleted gliomas. Decreasing strength with increasing anaplasia implied a modulatory effect. No somatic mutations were noted at this locus in 114 blood-tumor pairs, nor was there a copy number difference between risk-allele and only-ancestral allele carriers. CCDC26 RNA-expression was rare and not different between the two groups. There were only minor subtype-specific differences in common glioma driver genes. RNA sequencing and LC-MS/MS comparisons pointed to significantly altered MYC-signaling. Baseline enhancer activity of the conserved region specifically on the *MYC* promoter and its further positive modulation by the SNP risk-allele was shown *in vitro*. Our findings implicate *MYC* deregulation as the underlying cause of the observed association.

Single nucleotide polymorphisms (SNPs) at 8q24.21 have been associated with increased risk of IDH1/2-mutated gliomas^1^,^2^,^3^. A more recent study analyzed this region in more detail by pooled next-generation sequencing/imputation and identified a low-frequency SNP (rs55705857) that appeared very likely to be the causative-variant among several glioma-associated SNPs located at 8q24.21^4^. This finding was of great interest as the reported odds-ratio (OR) was the highest ever demonstrated for a genetic association with a human cancer. However, the genomic region where rs55705857 is located (8q24.21) contains no protein coding genes, no micro-RNAs and had no previously demonstrated mechanistic link to glioma development^5^,^6^. Nevertheless, the strict phylogenetic conservation of the region centered on rs55705857 in mammals and the exceptionally strong association with IDH-mutant gliomas suggested a functional role. The hypothesis of this study was that rs55705857 played a direct role in glioma oncogenesis and we sought clues by demographic-, clinical-, molecular-, transcriptomic- and proteomic- comparisons.

## Results

### rs55705857 is strongly associated with inherited glioma risk in the Turkish population

**Figure 1:**
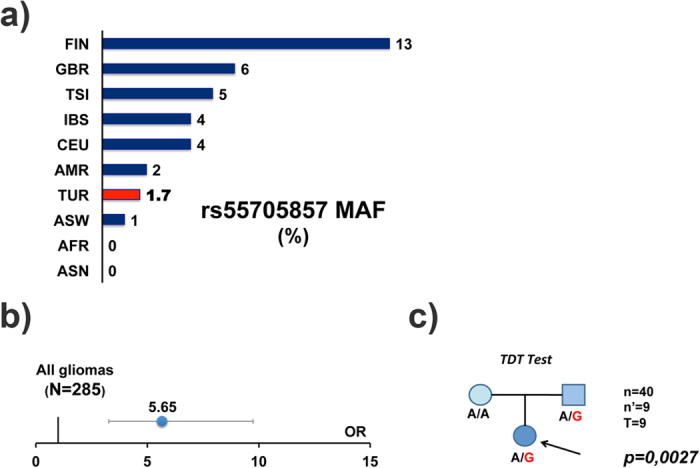
rs55705857 is associated with increased glioma risk in the Turkish population. (**a**) Comparison of rs55705857 G-allele frequency (MAF) in Turkish population to other populations. Allele frequency was obtained by genotyping a total of 727 controls (316 healthy controls and 411 cancer patients). (**b**) 285 glioma patients of various grades and pathologies were genotyped and odds ratio (OR) of developing glioma for rs55705857-G allele carriers was determined. Error bars indicate 95% confidence interval. (**c**) Trio based Transmission disequilibrium test (TDT) was employed to test for association between rs55705857 genotype and glioma risk. OR: odds ratio; p-value was calculated by chi-square-test.

**Figure 2:**
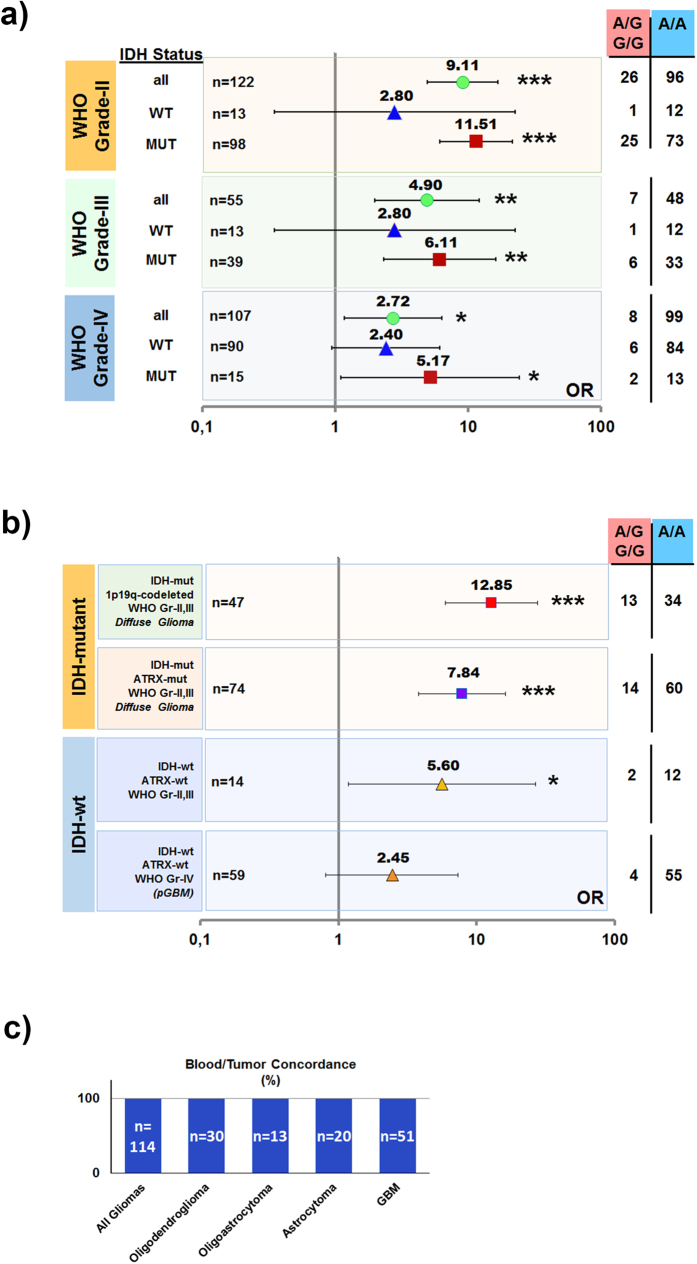
rs55705857 is associated with IDH mutations and lower-grade in gliomas. Stratification of gliomas by (**a**) grade and IDH mutation status, (**b**) molecular subtype that is based on IDH1/2 mutation, 1p/19q-codeletion, ATRX mutation status and grade. (square) indicates IDH-mutant tumors, (triangle) indicates IDH-wild-type tumors. Error bars indicate 95% confidence interval. Number of patients genotyped under each subtype is given on the left hand side and their genotype distribution on the right hand side, while the exact value of odds ratio is indicated above data points. (*) Indicates a statistical significance level of p < 0.05; (**) indicates a statistical significance level of p < 0.001; (***) indicates a statistical significance level of p < 0.0001. (**c**) 114 matched blood-tumor samples for genotyped for rs55705857 to exclude possible “Knudsonian second-hits”. 14 samples were of A/G genotype, 100 samples were of A/A genotype. y-axis indicates “%” concordance OR: odds ratio; n = number of patients.

Turkey has a diverse genetic makeup; therefore in order to confirm and recapitulate the previously reported association between rs55705857 and glioma-risk in the Turkish population, we performed a case-control experiment. DNA isolated from peripheral blood of 285 glioma patients, 316 healthy controls and 411 systemic cancer patients were genotyped (Supplementary Table 1). The minor allele frequency (MAF) of the G-allele was found to be 1.7% in the Turkish population, which is lower than that in European populations but higher than Asian and African populations (Fig. 1a). MAF in the glioma cohort was 7.5% and the Odds Ratio (OR) for all hemispheric diffuse glioma (DG) cases was 5.65 (%95 CI: 3.27–9.75; n = 285) (Figs 1b and 2). To exclude a type-1 error related to population heterogeneity, we performed transmission disequilibrium test (TDT) on 40 family trios (glioma patients and their healthy parents). The risk-allele was transmitted from one of the parents to the patient 9/9 times, with no incidence of A-allele being transmitted from a heterozygous parent to a patient (Fig. 1c), supporting the findings of our case-control study.

Next, we performed subgroup analysis of this association. We observed that the association was far stronger for IDH-mutant gliomas compared to IDH-wt gliomas (ORs of 9.25 vs. 2.49; p < 0.0001 vs. p = 0.033; n = 153 vs. 116, respectively) (Fig. 2a and Supplementary Table 2). Rs55705857-IDH association was strongest for WHO grade-II gliomas. IDH-mutant tumors across all histopathologic subtypes of lower-grade glioma (LGG, i.e. WHO grade-II and -III oligodendrogliomas, oligoastrocytomas and astrocytomas) were strongly associated with the risk-allele of rs55705857 (ORs of 10.55, 9.41 and 8.91; n = 67, 32, 38, respectively; all p < 0.0001) (Supplementary Table 2). There was only a marginally significant association between IDH-mutant GBM and rs55705857. On the other hand, an almost two-fold difference of rs55705857-associated risk between IDH-mutated WHO grade-II and grade-III tumors (ORs 11.51 vs. 6.11; p < 0.0001 vs. p = 0.0003, respectively) suggests a strong grade-specific bias of rs55705857-glioma association (Fig. 2a and Supplementary Table 2).

In accordance with previous reports^4^,^6^, the association was strong in oligodendrogliomas, regardless of the IDH-status of tumors (OR = 10.55 in IDH-mutant vs. 11.02 in IDH-wt tumors; p < 0.0001 vs. p = 0.0043; n = 67 vs. 8, respectively (Supplementary Table 2). In contrast, G-allele was absent from a total of 18 IDH-wt astrocytomas and oligoastrocytomas, compared to 2/8 (MAF = 12.5%) in IDH-wt oligodendrogliomas.

Next, we tested association of rs55705857 with molecular subtypes of gliomas. The strongest association was observed for the IDH-mutant, 1p/19q co-deleted, ATRX-wt, WHO grade-II or III DGs (OR = 12.85; p < 0.0001; n = 47) (Fig. 2b and Supplementary Table 2). The second strongest association was observed for IDH-mutant 1p/19q-intact ATRX-mut WHO grade-II or III DGs (OR = 7.84; p < 0.0001; n = 74). Another molecular group that seemed to be associated with the risk allele, albeit much weaker, was IDH-wt, ATRX-wt tumors (OR = 5.60; p = 0.0302; n = 14).

The exceedingly high ORs associated with the SNP prompted us to exclude the possibility of it rather behaving similar to a “mutation”. Screening of 114 tumor-blood pairs (14 (A/G) and 100 (A/A) patients) did not yield any “Knudsonian second hits” at the same locus (Fig. 2c).

### rs55705857 is not associated with increased risk in other 8q24.21-associated, IDH-associated or common systemic cancers

Several SNPs in the 8q24.21 locus have been associated with various cancer types including colorectal, prostate, lung, and breast cancers. Hence, we hypothesized that rs55705857’s effect on gliomas may not be specific to a possible involvement in 8q24.21-associated cancers. A total of 411 cancer patients’ peripheral blood DNA was genotyped and G-allele frequencies were compared to the healthy control population (Table 1). For all 411 cases combined, there was no association with rs55705857 (OR = 0.74, 95%CI = 0.33–1.78; p-value = 0.47). Colorectal (n = 46), breast (n = 69), lung (n = 55) and prostate (n = 32) cancers yielded ORs between 0.6 (colorectal) and 1.85 (prostate), but none reached statistical significance (p-values ranging between 0.43–0.81). We also tested a few of other systemic cancer types (e.g. pancreas, ovarian, stomach) and non-glioma CNS tumors (mostly meningiomas). G-allele frequency in other systemic cancers was 2.5% (n = 40; OR = 1.46; 95% CI = 0.31–6.83; p-value = 0.51). On the other hand, the G-allele was not detected in any of 31 CNS tumor cases tested (Table 1). Additionally, peripheral blood from 109 Acute Myeloid Leukemia (AML) patients and 34 other hematologic malignancies were also genotyped. The risk allele was present in 1 of 143 samples (OR = 0.19; 95% CI = 0.03–1.53; p = 0.12), resulting in a trend towards negative-association in hematological malignancies.

To exclude an association with IDH mutation rather than with IDH mutant gliomas, we screened other IDH-associated tumors. Besides gliomas, IDH1–IDH2 mutations are frequently observed in chondrosarcomas and cartilaginous tumors, some of them in patients with Ollier disease and Maffucci syndrome^7^,^8^. 73 chondrosarcoma (CHS) and 47 low-grade cartilaginous tumors (LGCT), all bearing IDH-mutations, from a British patient cohort, were screened and compared to other British controls (1000 Genomes Project, Ensembl database, www.ensembl.org). 17 of these were patients with Ollier syndrome, 2 of them with Maffucci syndrome, 1 with Coeliac disease and 100 were solitary tumors. None of the syndromic patients were rs55705857-G allele carriers and the MAF for all tumors was 4.80, compared to 6.59 for the British population (Table 2). Overall, there was no significant association between rs55705857 and IDH-mutant bone/cartilaginous tumors collectively (OR = 0.72; p = 0.46), CHS (OR = 0.99; p = 0.99) or LGCTs (OR = 0.32; p = 0.14; n = 47).

### Clinical behavior of tumors with and without rs55705857-G allele are not overtly different

Despite previous reports that linked rs55705857 with younger age^5^,^9^, our analysis of the age of onset failed to identify any statistically significant difference between risk allele carriers and non-carriers within the same glioma subtype (Supplementary Figure 1a). We compared risk allele carriers (A/G) to non-carriers (A/A) in WHO grade-II gliomas that were grouped according to either histopathology or molecular alterations. Based on WHO 2007 histopathologic classification, the median age for oligodendroglioma-A/G group (n = 12) was 36 (36.8 ± 1.6) compared to 38 (40.3 ± 1.5) for A/A group (n = 46) (values in parentheses are expressed as Mean ± S.E.M.). A similar trend was observed when molecular classification scheme was used: 37.5 vs. 38.0 (median), 40.0 ± 2.7 vs. 40.4 ± 2.0 (mean ± S.E.M.), n = 10 vs. n = 26, for oligodendrogliomas with A/G and A/A genotypes, respectively (Supplementary Figure 1a, left panel). One-way ANOVA, Kruskall-Wallis and Dunn’s multiple comparison tests did not reveal any significant difference between any of the groups tested. On the other hand, the picture was less clear with astrocytomas: Both histopathologic and molecular classification of IDH-mutant grade-II astrocytomas revealed a trend toward younger age of patients with A/G genotype, compared to those with A/A genotype; however, the difference did not reach the significance level of p < 0.05 (p = 0.22, Kruskall-Wallis test). The median and mean values of each group based on histopathology and rs55705857 were as follows: 29.0 vs. 34.0 (median), 31.0 ± 2.1 vs. 37.4 ± 2.1 (mean ± S.E.M.), n = 7 vs. n = 33, for astrocytomas with A/G and A/A genotypes, respectively (Supplementary Figure 1a, right panel). According to molecular classification: 30.0 vs. 34.5 (median), 31.9 ± 2.0 vs. 37.7 ± 1.9 (mean ± S.E.M.), n = 11 vs. n = 40, for patients with the A/G and the A/A genotypes, respectively.

A molecular marker that is indicative of proliferative capacity and therefore of clinical behavior of gliomas is Ki-67 mitotic index. We checked for any Ki-67 index differences in risk allele carriers through different histopathologies. Hence, we compared Ki-67 index distribution according to rs55705857 genotype and histopathologic/molecular classification criteria (Supplementary Figure 1b). In WHO grade-II oligodendrogliomas, median, mean and standard error values were as follows: 0.03 vs. 0.03 (median), 0.050 ± 0.0098 vs. 0.075 ± 0.015 (mean ± S.E.M.), n = 13 vs. n = 44, for the A/G and the A/A groups, respectively. Similar results were obtained with molecular grouping, i.e. IDH-mutant 1p/19q co-deleted ATRX-wt WHO grade-II DGs: 0.05 vs. 0.04 (median), 0.053 ± 0.0107 vs. 0.076 ± 0.0169 (mean ± S.E.M.), n = 10 vs. n = 26, for the A/G and the A/A groups, respectively. Similarly, analysis of WHO grade-II astrocytomas yielded no significant difference between the two genotypes, either: 0.04 vs. 0.03 (median), 0.036 ± 0.0078 vs. 0.034 ± 0.0046 (mean ± S.E.M.), n = 7 vs. n = 32, for the A/G and the A/A groups, respectively. Neither did it among IDH-mutant 1p/19q-intact ATRX-mut WHO grade-II DGs: 0.02 vs. 0.03 (median), 0.026 ± 0.0036 vs. 0.043 ± 0.012 (mean ± S.E.M.), for A/G and A/A groups, respectively. One-way ANOVA, Kruskall-Wallis and Dunn’s multiple comparison tests were applied for statistical analysis of all groups and the p-value was found to be p = 0.84. Therefore, we conclude that there is no association between rs55705857 genotype status and Ki-67 index.

In order to assess the impact of the presence of rs55705857-G allele on the clinical progression of tumors, we compared “time to malignant degeneration” (Supplementary Figure 1c) and “overall survival” (Supplementary Figure 1d) of WHO grade-II IDH-mutant 1p/19q co-deleted oligodendrogliomas and IDH-mutant astrocytomas with either the A/G or A/A genotype. Kaplan-Meier curve analysis and log-rank test did not reveal any observable difference among WHO grade-II IDH-mutant astrocytomas in terms of overall survival or time to malignant degeneration (p = 0.479 and p = 0.883, respectively). On the other hand, among IDH-mutant 1p/19q co-deleted oligodendrogliomas, a trend towards both longer survival and longer time to malignant degeneration was evident for A/G patients; however, it did not reach the 95% statistical significance level (p = 0.234 and p = 0.281, respectively).

### Oncogenic changes in relation to rs55705857

To test for any further association other than the IDH-mutation we compared risk allele carriers with non-carriers for common glioma associated oncogenic changes (*IDH1/2* and *TERT*-promoter mutations, and immunohistochemistry for ATRX, TP53, p16^INK4A^, PTEN, EGFR and *MGMT* methylation). Among them, *TERT* promoter (TERTp) mutations C228T and C250T were recently described in gliomas and a possible relation with rs55705857 has never been explored so far^10^.

**Figure 3:**
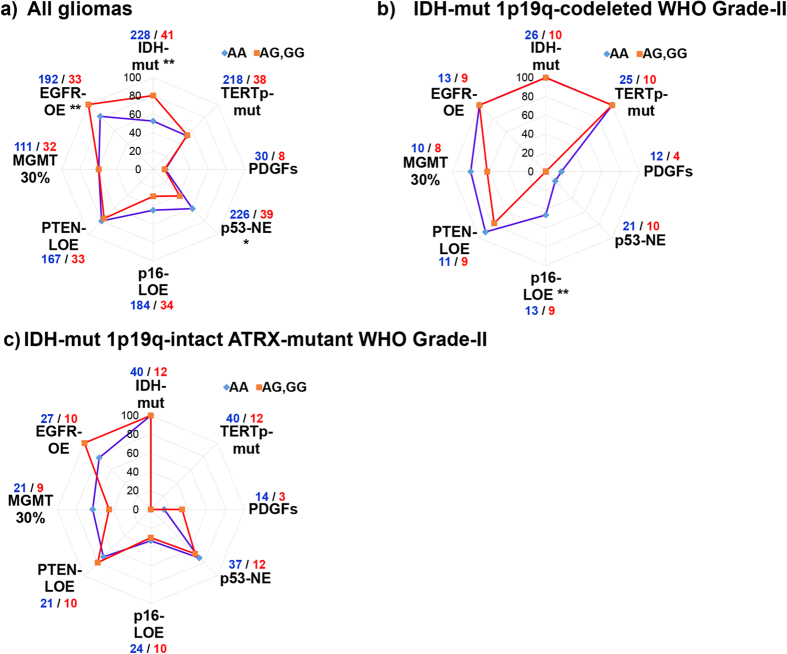
Distribution of molecular alterations according to rs55705857 genotype and glioma subtype suggests subtype-specific differences. Differential distribution of molecular alterations between A/G and A/A groups of (**a**) all gliomas, (**b**) WHO grade-II oligodendrogliomas with IDH mutation, 1p/19q-codeletion and lack of ATRX mutations, (**c**) WHO grade-II astrocytomas with IDH mutation and ATRX mutations, were plotted as radar graphs. ATRX, p53, p16, PTEN, EGFR and *MGMT* status was determined by immunohistochemical staining of FFPE-sections. An arbitrary level of 30% was used to determine *MGMT* promoter methylation. *IDH1/2* and *TERT* promoter mutations were detected by PCR and Sanger sequencing of target region or by “mini-sequencing”. PDGFs indicates copy number gain in any of PDGFA, PDGFB, PDGFRA or PDGFRB, determined by exome sequencing. Number of samples analyzed for each alteration are indicated at the corners of the radar graph in blue (A/A) and red (A/G, G/G). Differential distribution of each molecular alteration between A/G and A/A groups of tumors was determined by chi-square test. y-axis indicates percentage of samples with alteration. (*) Indicates a significance level of p < 0.05; (**) indicates a significance level of p < 0.01. LOE: loss-of-expression; NE: nuclear-expression; TERTp: *TERT* promoter.

Rs55705857-G allele showed the strongest association with IDH1 mutation in “all gliomas” group (53% vs. 81%, for A/A and A/G, respectively; p = 0.001, determined by chi-square test) (Fig. 3a). In the same group, the second strongest association was with EGFR overexpression, as determined by immunohistochemistry (82% vs. 100%, for A/A and A/G, respectively; p = 0.0076). A third marker that reached significance in “all gliomas” group was p53 nuclear expression (p53-NE). p53 protein has a very short half-life of 5–20 min and widespread nuclear accumulation of p53 is not observable in normal tissues; it is indicative of mutations in the p53 pathway. Loss-of-p16^INK4A^ expression (p16-LOE) frequency was not statistically different between A/G and A/A group tumors. On the other hand, frequency of TERTp-mutations (52% vs. 53%), loss-of-PTEN expression (PTEN-LOE, 80% vs. 76%), copy number gain of PDGFs (PDGFA, PDGFB, PDGFRA, PDGFRB; 13% vs. 13%) and *MGMT* promoter methylation above an arbitrary 30% level (59% vs. 59%) was almost identical between A/A and A/G groups (Fig. 3a).

As gliomas are a molecularly heterogeneous group, we narrowed down our analysis to molecularly similar diffuse gliomas: We analyzed *IDH*-mutant 1p/19q co-deleted ATRX-wt WHO grade-II and *IDH*-mutant 1p/19q-intact ATRX-mut WHO grade-II DGs, separately, and looked for possible associations between molecular alterations and the rs55705857 genotype. Interestingly, we noted that p16^INK4A^-LOE was observed in 6 out of 13 (46%) *IDH*-mutant 1p/19q co-deleted ATRX-wt WHO grade-II DGs with the A/A genotype but in none of those with the A/G genotype (n = 9, p = 0.009) (Fig. 3b). None of the other markers were differentially distributed between the two genotypes of *IDH*-mutant 1p/19q co-deleted ATRX-wt WHO grade-II DGs. As opposed to the “all gliomas” group, EGFR was overexpressed in all tumors (100%) of *IDH*-mutant 1p/19q co-deleted ATRX-wt WHO grade-II regardless of the rs55705857 genotype. On the other hand, in the *IDH*-mutant 1p/19q-intact ATRX-mut WHO grade-II DG group, the only marker that approached statistical significance was EGFR overexpression: it was present in 10/10 *IDH*-mutant 1p/19q-intact ATRX-mut WHO grade-II DGs of the A/G genotype and 21/27 of the A/A genotype (p = 0.10 by chi-square test) (Fig. 3c). Frequency of p53-NE, p16^INK4A^-LOE, PTEN-LOE and copy number gain of PDGFs (PDGFA, PDGFB, PDGFRA, PDGFRB) were similar between the two groups.

Although investigation of possible correlations between rs55705857 and well-known molecular changes in gliomas provides valuable information, a mechanistic explanation necessitates an understanding of its function. Therefore, we hypothesized that rs55705857 may affect the expression of the *CCDC26* lncRNA gene that it resides within. Quantitative real-time PCR (qPCR) analysis with a TaqMan assay that targets the common exons (exons 3–4) of *CCDC26* did not yield a significant difference between the G-allele carriers and non-carriers (1/10 vs. 4/28 of samples with detectable expression, respectively; chi-square test p-value = 0.73). This result indicates that the G-allele is unlikely to exert its impact by altering *CCDC26* expression. However, we cannot rule out possible effects on alternative transcripts that do not contain exons 3 or 4.

The 8q24 locus is commonly amplified in gliomas and an obvious question is whether rs55705857 has any association with copy number variations (CNV) at this locus or elsewhere in the genome. Whole genome CNV analysis was performed in 22 WHO grade-II *IDH*-mutant oligodendrogliomas, A/A (n = 18) and A/G (n = 4) genotypes, using OncoScan FFPE Express arrays. These arrays are based on molecular inversion probe technology and have the advantage of detecting copy-neutral events in addition to gains/losses. None of the 4 tumors in the A/G group had any CNV at 8q24, while 2/18 (11%) tumors in the A/A group had copy number gain (p = 1.00 by Fisher’s exact test) (Supplementary Figure 2 and Supplementary Table 3). These findings suggest that rs55705857 is unlikely to be associated with mechanisms that affect 8q24.21 copy number or require copy-number changes to show its effects.

### Network analysis of transcriptome data by PANOGA suggests differential neurotrophin and Ras/MAPK signaling

Candidate based analysis of molecular alterations hinted at a differential involvement of certain genes/pathways in tumors with rs55705857-G allele compared to those with A-allele. However, only limited information could be gathered with this type of candidate based approach. Therefore, we next undertook transcriptomic analysis of tumors followed by network analysis by PANOGA (Pathway and Network Oriented GWAS Approach)^11^.

To this end, we performed RNA-seq analysis of 6 WHO grade-II tumors (n = 4 with the A/G, n = 2 with the A/A genotype) that were *IDH1*-R132H mutant, 1p/19q co-deleted and ATRX-wild-type. By choosing tumors that are low-grade and highly similar to each other in terms of molecular alterations, we aimed at minimizing the confounding factors and maximize the chances of identifying differences that are associated with rs55705857 genotype. Another important aspect of the strategy that we used to maximize differences due to rs55705857 and minimize the others was to compare tumors-to-tumors (i.e. A/G to A/A), rather than comparing tumors to the “normal” brain tissue. Our approach by-passed the problem of “normal tissue”, as all attempts at using various sources of brain tissue as “normal control” make untestable assumptions that can lead to false conclusions.

A total of 72 genes were found to be differentially expressed at p ≤ 0.001 and q < 0.2 significance level between the two groups (Supplementary Table 4). Network analysis of RNA-seq data was carried out by a modified PANOGA^11^. Originally developed for network analysis of GWAS data, PANOGA combines evidence from a variety of sources and extracts information not only from relatively few highly significant genes but also accounts for contribution of numerous other less-significant genes. Therefore, 822 genes differentially expressed at p < 0.05 level were analyzed by a modified PANOGA approach. The results suggested “neurotrophin signaling pathway”, “Ras/Rap1/MAPK pathways”, “osteoclast differentiation pathway” as the most significant KEGG pathways being differentially regulated between WHO grade-II *IDH*-mutant 1p/19q co-deleted DGs with rs55705857-A/G and rs55705857-A/A genotypes (Supplementary Table 5). Less significant pathways included several related to immune response and cell adhesion related processes.

### Proteomic analysis identifies differential MYC network activity

**Figure 4:**
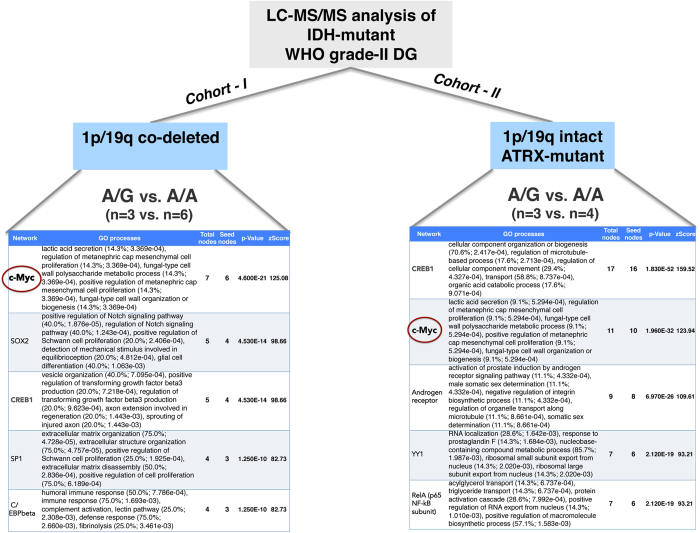
Transcriptional regulation analysis of LC-MS/MS comparison of WHO grade-II oligodendrogliomas and astrocytomas point to differential MYC network activity between rs55705857-G allele carriers and non-carriers. **Left panel:** MetaCore transcriptional regulation analysis of differentially expressed proteins between A/G (n = 3) and A/A (n = 6) groups of IDH-mutant 1p/19q co-deleted ATRX-wt diffuse gliomas. **Right panel:** the same analysis was performed on LC-MS/MS data of IDH-mutant 1p/19q-intact ATRX-mut diffuse gliomas, A/G (n = 3) and A/A (n = 4) groups. “GO processes” and the particular p-value for each process is listed in the second column of each panel. zScore indicates the standard/normalized score for each transcription factor network analyzed.

Next, we extended our integrative approach to include proteomic analyses of two distinct molecular subtypes of WHO grade-II gliomas, namely *IDH*-mutant 1p/19q co-deleted ATRX-wt WHO grade-II DGs (n = 3 vs. n = 6, A/G and A/A, respectively) and *IDH*-mutant 1p/19q-intact ATRX-mut WHO grade-II DGs (n = 3 vs. n = 4, A/G and A/A, respectively). LC-MS/MS analysis of tumors yielded a total of 1561 proteins that were identified with at least 2 peptides at an FDR < 0.1% significance level. 15 proteins were differentially expressed among *IDH*-mutant 1p/19q-intact ATRX-mut WHO grade-II DGs, while 35 proteins were differentially expressed among *IDH*-mutant 1p/19q co-deleted ATRX-wt WHO grade-II DGs with and without the G-allele (Supplementary Tables 6 and 7). However, enrichment analysis of these proteins by MetaCore^TM^ (GeneGo Inc., Thomson Reuters) did not yield any pathway or GO process with an FDR < 10^−5^ significance level. Therefore, we decided to look for possible transcription factor (TF) networks that are differentially affected by rs55705857 alleles, by using “transcriptional regulation analysis” function of MetaCore. MYC transcriptional network was the top hit in *IDH*-mutant 1p/19q co-deleted ATRX-wt WHO grade-II DGs (p-value 4.6E-21) and the second top hit in *IDH*-mutant 1p/19q-intact ATRX-mut WHO grade-II DGs (p-value 1.96E-32) (Fig. 4). The second most significant TF was *CREB1* (cAMP Response Element Binding Protein 1) – top hit in *IDH*-mutant 1p/19q-intact ATRX-mut WHO grade-II DGs (p-value 1.83E-52) and third top hit in IDH-mutant 1p/19q co-deleted ATRX-wt WHO grade-II DGs (p-value 4.53E-14). Of note, GO processes associated with both transcription factor networks were nearly identical between molecular oligodendrogliomas and astrocytomas, implying common processes are affected in both types of gliomas in the presence of the G-allele.

Considering the well-known roles of *MYC* in various cancer types including gliomas and its relative proximity to rs55705857 on chromosome 8, we hypothesized that rs55705857 may modulate *MYC* expression.

### A conserved region encompassing rs55705857 is an enhancer of *MYC* promoter activity

Several SNPs in 8q24.21 region that increase cancer risk have been shown to do so by long-range interactions with *MYC* proto-oncogene promoter in a tissue specific manner^12^,^13^,^14^. Moreover, some of these SNPs have recently been shown to interact with *CCDC26* locus in breast and prostate cancer cells, as well^15^,^16^. Studies on AML and ALL suggested that 100 kb region flanking rs55705857 contains 5 tissue-specific *MYC* enhancers and, despite being 1.9 Mb apart from *MYC* gene, interacts with SWI/SNF chromatin remodeling complex subunit Brg1 (in AML) or transcription factor Tal1 (in ALL)^17^,^18^,^19^. Collectively, these findings suggest a possible regulatory function for the region encompassing rs55705857.

**Figure 5:**
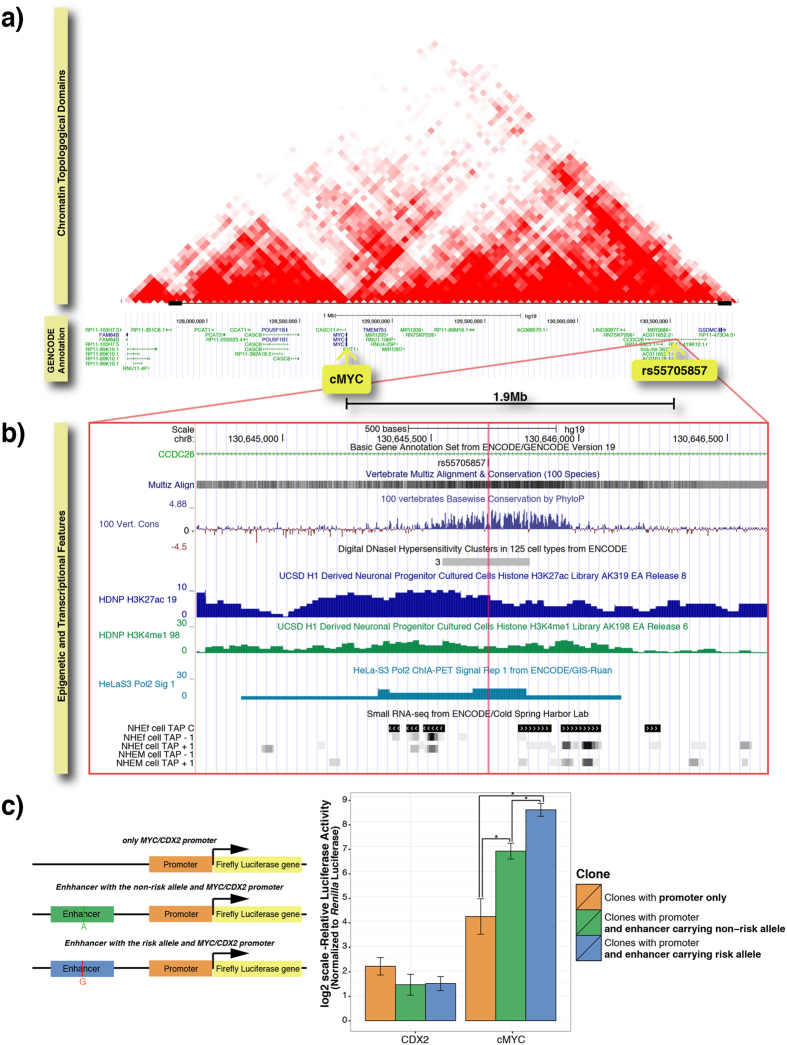
The region encompassing rs55705857 acts as a *MYC* enhancer and rs55705857-G allele increases this activity. (**a**) Chromatin Topological Domain structure and epigenetic and transcriptional features of the region around rs55705857 and *MYC*. Normalized Hi–C interaction frequency displayed as a two-dimensional heat map showing the topologic domain that includes the glioma risk allele rs55705857 as well as the *MYC* oncogene (Adapted from^21^). The frequency of interaction between two 40-kb genomic regions is indicated by the color intensity at their diagonal intersection. (**b**) Epigenetic and transcriptional features, obtained from UCSC Genome Browser, are as follows 1) The genomic region encompassing rs55705857 shows evidence of a high sequence conservation between evolutionarily-divergent species, 2) rs55705857 resides in DNaseI hypersensitivity site, detected in 3 cell-lines (one of which being hippocampal astrocyte cell line HA-h), 3) This genomic region displays H3K27ac and H3K4me1 signals, both of which distinguish active enhancers from inactive/poised enhancer elements, detected in H1-derived neuronal progenitor cells, 4) An RNA polymerase II binding site, as indicated by Pol2 ChIA-PET signal in HeLaS3 cells, overlaps the genomic region encompassing rs55705857, suggesting active-transcription, 5) This genomic region also displays a balanced short RNA transcription, characteristic of enhancer-associated RNAs (eRNAs), which is a hallmark of active enhancers. (small RNA-seq data). (**c**) The left panel shows the organization of luciferase assay constructs. HEK293 cells were transfected with Renilla luciferase plasmid pRL-CMV and one of three firefly luciferase plasmids: *MYC* promoter alone, *MYC* promoter plus enhancer region with rs55705857-A allele or *MYC* promoter plus enhancer region with rs55705857-G allele. As a control, *MYC* promoter was replaced with the *CDX2* promoter and assayed in parallel. After 48–72 hours, luciferase expression was measured via Dual-Glo Luciferase Assay System. Average of 3 experiments are shown (errors bars indicate S.E.M). y-axis shows normalized luciferase activity in logarithmic scale. (*) Indicates a statistical significance level of p < 0.05.

Using VISTA Enhancer Browser^20^, a resource that examines numerous potential enhancer elements *in vivo* with transgenic mouse assays, rs55705857 was shown to overlap the human enhancer element, hs1709. Moreover, this region is part of a single chromatin topologic domain (Fig. 5a^21^) that contains the *MYC* oncogene, which serves as the most likely candidate target gene for this regulatory element.

Conservation of non-coding sequences among divergent species has been suggested to be a good predictor of cis-regulatory elements. In addition, variation within these conserved regions has been proposed to be associated with phenotypic differences. The genomic region encompassing rs55705857 showed evidence of high sequence conservation between evolutionarily-divergent species, with a GERP score 5.98.

To infer a possible regulatory function, we assessed regulatory features using UCSC Genome Browser, revealing that rs55705857 overlaps a DNase I hypersensitivity site (one of the three cell lines with hypersensitivity is HA-h, a hippocampal astrocyte line), histone marks that are associated with regulatory elements in H1-derived neuronal progenitor cultured cells, a RNA-Pol2 binding signal, and small RNA-seq data showing a balanced short RNA transcription, characteristic of enhancer-associated RNA (eRNA), a hallmark of active enhancers (Fig. 5a; Data obtained from the UCSC Genome Browser https://genome.ucsc.edu/). All these features suggest that rs55705857 may be a regulatory SNP, residing in a cancer-specific enhancer element.

To test for this possible enhancer activity on *MYC* and determine any allelic difference, we performed luciferase reporter assay in HEK293 cells. Three different firefly luciferase constructs were prepared, two containing the highly conserved enhancer element cloned upstream of *MYC* promoter, with the only difference between the two being the presence of risk or non-risk allele, and one with only the *MYC* promoter (Fig. 5c).

Both constructs with the enhancer element displayed a higher firefly luciferase activity (normalized to *Renilla* luciferase activity) than the construct with the *MYC* promoter alone, supporting the enhancer activity hypothesis of the conserved region. More importantly, the construct containing the risk allele displayed a significantly higher (approximately 3 times) luciferase activity than the construct with the non-risk allele, suggesting that the presence of the risk allele causes a stronger transcriptional enhancing activity on *MYC* promoter (Fig. 5c). On the contrary, the enhancer activity was observed only in combination with the *MYC* promoter, as no increase in the activity of the *CDX2* promoter was observed. Moreover, the presence of the risk allele or non-risk allele did not make any difference when combined with the *CDX2* promoter. These results indicate that the conserved region around rs55705857 can enhance the transcriptional activity of the *MYC* promoter specifically and the risk allele further augments this effect.

Although multiple lines of evidence we obtained showed that rs55705857 is associated with altered MYC activity, we did not find difference in MYC protein expression by immunohistochemical staining of 31 LGG samples (n = 21 vs. n = 10, for A/G and A/A, respectively; Supplementary Figure 3a). Neither did we observe any difference between the two groups in terms of copy number gain of MYC (Supplementary Figure 3b) or any oncogenic copy number alteration of the MYC-MAX-FBXW7 module (Supplementary Figure 3c). A plausible explanation is that the effect exerted by rs55705857 shifts *MYC* expression only modestly and possibly only in a certain cell type, such as glioma initiating cells. Indeed, a study that investigated the effects of the deletion of a *MYC* enhancer that is implicated in colorectal cancer showed that the deletion led to cancer resistance while having only slight or no impact on *MYC* expression^22^. Therefore, a more detailed and extensive analysis may be required to accurately measure the effect of rs55705857 on *MYC* expression.

## Discussion

Our study has three main findings: First, the rs55705857-associated glioma risk was confirmed in the Turkish population. Second, the association was found to be unique to *IDH*-mutant gliomas (but not gliomas in general, other central nervous system malignancies, other common systemic cancers, other 8q24.21-associated systemic cancers or IDH-mutant non-glioma tumors). Third, the evolutionarily conserved region encompassing rs55705857 may act as a *MYC*-enhancer and the presence of the risk allele further potentiates this activity.

We found the minor allele frequency (MAF) for rs55705857 as 1.7% in the general Turkish population and 7.5% in hemispheric adult gliomas. Confirming previous findings, the association was 4-times stronger for *IDH*-mutant gliomas when compared to IDH-wt cases in our cohort^4^,^6^. The association with *IDH*-mutant gliomas was persistently significant, when analyzed for different WHO-grades or for different genetic subtypes (carrying mutational profiles attributed to oligodendrogliomas or astrocytomas by the 2014 Haarlem Consensus)^23^. In contrast, there was never a significant association with *IDH* wild-type gliomas. Given the fact that *IDH*-mutant gliomas peak at the 4^th^ and 5^th^ decades of life and that the *IDH* wild-type gliomas present with an ever-increasing incidence with age, independent oncogenic processes may be active in *IDH*-mutant and wild-type gliomas. Therefore, rs55705857 may only be interfering with the oncogenic process in *IDH*-mutant gliomas.

Even though evidence strongly suggests a causal role for rs55705857 in gliomagenesis, the fact that only a tiny fraction of rs55705857-G allele carriers develop glioma points to a modulatory role for this SNP. Considering that regulation of *MYC* expression is under control by several factors and mechanisms, it would not be surprising to see that alteration of only one of those mechanisms to result in a limited effect on the phenotype. In theory, such a minor, modulatory effect shall become less and less significant in the context of an ever-increasing anaplasia with accumulating oncogenic properties. In *IDH*-mutant gliomas, we noted such a decrease in the degree of significance with increasing anaplasia (WHO grade), the mechanism of which is yet to be elucidated. Our speculation that rs55705857 marks less malignant cases is supported by findings from clinical studies, which showed that rs55705857 is a marker of longer progression-free survival in patients with oligodendrogliomas^24^. In our patient cohort, the carriers of the risk allele at rs55705857 had a trend towards longer “overall-survival” or longer “time to malignant degeneration” in oligodendrogliomas but not in astrocytomas. Other important predictors of clinical behavior such as patient age at presentation or the Ki-67 proliferative index were not statistically different in carriers and non-carriers of the risk allele.

The association of rs55705857 with *IDH*-mutant gliomas is the strongest reported so far for any cancer and the minor allele frequency is very low. Therefore, to exclude the possibility that it is not merely a polymorphism but a germline oncogenic mutation, we also genotyped tumor tissues from the same patients but no further somatic changes were found in the tumor tissue (n = 114). The small number of cases homozygous for the risk allele (n = 2; 4.4% of risk allele carriers) did not allow a useful comparison for the effect of gene dosage in heterozygous vs. homozygous carriers.

8q24 is a gene-poor region, but despite this fact it has several well-documented associations with multiple systemic cancers including prostate, colorectal, breast, bladder and lung in addition to gliomas^25^,^26^,^27^,^28^. To exclude a general cancer predisposition related to rs55705857, we tested several systemic cancers including lung, colorectal, breast and prostate but a possible association with rs55705857 was excluded for all (n = 411) or each cancer type individually. Similarly, we excluded a rather general association with *IDH*-mutation by testing 120 *IDH*-mutant CHS/LGCT patients. On the contrary, there was a trend towards a “protective effect” in hematologic malignancies and LGCT. This may be of importance as *IDH*-mutations denote reciprocal clinical behavior in gliomas (better survival) and *IDH*-mutant acute myeloid leukemia (AML) cases (worse survival)^29^,^30^.

Subtype specific associations of rs55705857 with loss of p16^INK4A^ expression, EGFR overexpression and p53 nuclear accumulation suggests that the G-allele may increase tumorigenic potential of tumor initiating cells through partially different routes in oligodendrogliomas and astrocytomas. Overexpression of EGFR in 100% of oligodendrogliomas with the G-allele may be related to differential activity of Ras/MAPK pathways and point to possible dependence on RTK activation in these tumors. Despite differences in molecular alterations associated with the risk allele, MYC network alteration was shared between both oligodendrogliomas and astrocytomas as one of the most significant.

Association of gliomas with 8q24.21 has been reported for different SNPs (e.g. rs4295627, rs891835), but an underlying mechanism has not been reported for any of them. With rs55705857, the first plausible explanation would be an effect on local gene expression, namely of *CCDC26* (RAM). The expression of this lncRNA has first been reported in leukemia/lymphoma cell lines. However, *CCDC26* has never been reported to be expressed in gliomas. In agreement, our quantitative analysis using qPCR (n = 38) and RNA-seq methods (n = 7) did not detect significant expression of *CCDC26* in the majority (90%) of glioma tumor samples, which indicated that rs55705857 is unlikely to exert its effect by modulating the expression of major isoforms of *CCDC26* in tumor cells.

Some of the SNPs at the 8q24 locus that had been linked to systemic cancers including prostate, colorectal and breast were shown to have long-distance interactions with the *MYC* promoter and regulate its expression in a tissue-specific manner^12^,^13^,^14^. So far, no mechanistic link has been demonstrated in gliomas between rs55705857 and *MYC*, which is located approximately 1.9 Mb centromeric to rs55705857 (as opposed to aforementioned systemic cancer associated-SNPs, which are situated centromeric to *MYC*). Immunohistochemical staining of a relatively small group of gliomas did not reveal any detectable difference in MYC expression in carriers and non-carriers of the risk allele; however, comparative LC/MS-MS analyses, when performed separately in oligodendroglioma and astrocytoma tumor samples independently, consistently indicated that the MYC-network was the most differentially regulated transcriptional network in risk-allele carriers with high significance. Observation of the same effect in two different histologies, both of which are IDH-mutation driven, further supports this *MYC* dependent explanation of the 8q24.21-association in gliomas. Furthermore, *in vitro* luciferase assays showed that the genomic region centered on rs55705857 has a positive modulatory effect on the *MYC* promoter and that the presence of the risk allele significantly augmented this already positive modulation. The fact that *MYC* does not function as an on-off switch, but rather acts as a continuous modulator is also consistent with the modulatory effect of the rs55705857. Extrapolations from the age of onset and from the growth rate of IDH-mutant low-grade gliomas place the onset of glioma development to early childhood or the in-utero period, which are active times of neurogenesis and gliogenesis. *MYC* is an important player in cortical development, and is an established key-effector in the gliogenesis-neurogenesis switch^31^. A recent report showed that elevated *MYC* expression in oligodendrocyte precursor cells (OPCs) keeps these cells in undifferentiated state and promotes their proliferation by regulating expression of cell cycle and nucleosomal genes^32^. Several possible roles for *MYC* in gliomagenesis have been documented, among which being a regulator in glioma stem cells and neural stem cells is of particular importance^33^,^34^. PTEN and p53 were suggested to regulate *MYC* and thereby regulate stem cell behavior in glioma^35^. Interestingly, mice that overexpress *MYC* in astroglia are more prone to gliomas^36^, and *MYC* has even been considered as a therapeutic target in gliomas^37^. We speculate that rs55705857 increases *MYC* expression in a particular spatial and temporal context and therefore increases the probability of tumor formation. Since the risk allele is an inherited factor, it is possible that more than one cell type is affected and contributes to gliomagenesis. On the other hand, the uniqueness of the association to gliomas and its absence from other *IDH*-mutated tumors may be explained by the enhancer being in an active state only in certain cell types. DNase I hypersensitivity of the region in only 3 out of 125 cell lines supports this idea. Further experiments will be required to test this hypothesis.

Studies on induced pluripotency have indicated that MYC is only transiently required during establishment of pluripotency and silenced afterwards^36^. Therefore, rs55705857 mediated increase in *MYC* expression do not have to be sustained through all stages of gliomagenesis and instead may very well be largely restricted to a certain cell type (e.g. OPCs or NSCs) and/or to a specific stage; this may be the reason behind why increased MYC activity cannot be shown simply and directly by *MYC* expression. In line with this hypothesis, the 8q24 localized and prostate cancer associated SNP (rs6983267) was shown to increase *MYC* expression only during prostate development/maturation before the onset of tumorigenesis^13^. A similar impact may be exerted by rs55705857 during brain development and/or in NSCs/OPCs.

MYC has long been known to cooperate with Ras at multiple levels *en route* to tumorigenesis^38^,^39^,^40^. Therefore, it is not surprising that PANOGA analysis of RNA-seq results identified Ras/MAPK pathways among the most significantly altered networks between risk-allele carriers and non-carriers. Knock-down of Rap1 has been identified to be synthetic lethal to *MYC* overexpression in human mammary epithelial cells (HMECs)^41^ Despite being altered in many different cancer types, deregulation of MYC expression could be toxic to cells and induce apoptosis^42^. Therefore, it must be critical for tumor initiating cells to activate compensatory mechanisms in parallel to *MYC* activation.

The second most affected transcriptional network, particularly in IDH-mutant 1p/19q co-deleted ATRX-wt WHO grade-II DGs, was that of CREB1 (cAMP response element binding protein 1), a well-known mediator of neuronal processes including neurogenesis. CREB is also a classical mediator of neurotrophin response in neurons and may explain differential regulation of neurotrophin pathway in gliomas with and without rs55705857-G allele^43^. Recently, CREB phosphorylation has been inversely associated with 1p/19q-codeletion, the hallmark alteration of oligodendrogliomas^44^. However, the molecular machinery that links rs55705857 to altered CREB network is the subject of further studies.

## Methods

### Genotyping of patients and controls

Peripheral blood was collected from patients and controls after informed consent was obtained. Blood was collected into EDTA-tubes and genomic DNA was extracted with High Pure PCR Template Preparation Kit (Roche) or MagNA Pure LC DNA isolation kit on MagNA Pure LC 2.0 automated system (Roche, Mannheim, Germany). DNA isolation from FFPE-sections, tumor samples stored in liquid nitrogen or RNAlater (Life Technologies) was performed with QIAMP DNA Mini Kit (QIAGEN, Hilden, Germany). FFPE-tissues were treated with xylene/ethanol before DNA isolation. See Supplementary Information for detailed description of sequencing protocols and primers.

### Immunohistochemical, cytogenetic and mutational characterization of tumors

Tumor samples were evaluated by one single neuropathologist and immunohistochemistry (IHC) was performed with antibodies against ATRX (Cat.# HPA001906, Sigma-Aldrich, Steinheim, Germany), p53 (Clone# DO-7, Cat.# A00021-IFU, ScyTek Laboratories, Logan, UT), PTEN (Clone# 17.A, Cat.# MS-1601, Thermo Fisher Scientific, Fremont, CA), p16^INK4A^ (Clone# E6H4, Cat.# 725-4713, CINtec p16 Histology, Roche Ventana, Basel, Switzerland), EGFR (Clone# EGFR.113, Cat.# NCL-L-EGFR, NovocastraTM Liquid Mouse Monoclonal Ab, Leica Biosystems Newcastle Ltd., Newcastle, UK), IDH1-R132H (Clone# H09, Cat.# DIA-H09, Dianova GmbH, Hamburg, Germany), Ki-67 (Clone# MIB-1, Cat.# M7240, Dako Denmark A/S, Glostrup, Denmark), MYC (Clone# Y69, Cat.# 790-4628, Roche Ventana, Basel, Switzerland) and methylated *MGMT* (Clone# MT23.2, Cat.# NB100-168, Novus Biologicals, Cambridge, UK), as described before^45^. An arbitrary 30% cut-off level was used for determination of *MGMT* methylation. 1p/19q-codeletion status was assessed by FISH and/or microsatellite analysis. Fluorescent PCR and fragment analysis by capillary electrophoresis of D1S162, D1S199, D1S226, D1S186, D1S312, D19S112, D19S918 and D19S206 STR regions for detection of loss of heterozygosity (LOH) in 1p and 19q in the tumor tissue was performed in ABI PRISM^®^ 3100 Genetic Analyzer (Applied Biosystems, ThermoFisher, Carlsbad, CA)^46^. All samples underwent routine IDH testing by IHC (anti-IDH1-R132H) and by molecular methods (mini-sequencing and/or Sanger sequencing). (Supplementary Information).

### Sample preparation for proteomics analysis

Tumor samples that have been flash frozen in liquid nitrogen were homogenized (Daihan Scientific) and lysed on ice. Homogenization buffer contained 50 mM ammonium bicarbonate (Sigma), 8 M urea (Sigma-Aldrich), 2 M thiourea (Sigma-Aldrich), Complete protease inhibitor cocktail tablets (Roche), and Phosstop phosphatase inhibitor cocktail tablets (Roche). Lysed tissue samples were centrifuged at 5000 g for 20 minutes at 4 °C. Supernatant aliquots were stored at −20 °C until LC-MS/MS analyses.

Protein extracts were reduced with 5 mM dithiothreitol (Sigma-Aldrich, USA) for 30 min at 60 °C in 0.05 M ammonium bicarbonate (Sigma-Aldrich, USA), pH 7.8 followed by an alkylation with 15 mM iodoacetamide (Sigma-Aldrich, USA) for 30 min at room temperature in the dark. After quenching the reactions with 15 mM dithiothreitol, proteins were digested with trypsin (Promega, Madison, WI) overnight at 37 °C to a final enzyme:substrate ratio of 1:50. The digestion was stopped by adding formic acid to a final concentration of 1%. Peptide mixtures were desalted using Finisterre SPE C18 reverse phase cartridges (Wicom International AG, Maienfeld, Germany) according to the following procedure: wetting the cartridge with 1 volume of 100% methanol, washing with 1 volume of 80% acetonitrile, equilibrating with 4 volumes of 0.1% formic acid, loading acidified digest, washing 6 volumes of 0.1% formic acid, and eluting with 1 volume of 50% acetonitrile in 0.1% formic acid. Peptides were dried using a vacuum centrifuge and resolubilized in 0.1% formic acid and frozen at −20 °C.

### Cloning, transfection and luciferase Assay

The 2774-bp MYC promoter fragment was amplified from genomic DNA extracted from the blood sample of a patient. The region was amplified via PCR with the primers MYCP_F1 (CGTCTATGTACTTGTGAATTATTTCA) and MYCP_R (*GGGGACCACTTTGTACAAGAAAGCTGGGTC*CTAAGCAGCTGCAAGGAGAGCCTTT), and then a nested PCR using the product of the first reaction as a template with the primers MYCP_F2 (*GGGGACAAGTTTGTACAAAAAAGCAGGCTTC*GCCATTACCGGTTCTCCATAGGGTG) and MYCP_R, which had flanking attB sites. The final products were separated on an agarose gel, and the desired bands were excised and purified, using the QIAquick Gel Extraction Kit (QIAGEN, Hilden, Germany). The purified product was then recombined into the pDONR221 plasmid via a Gateway BP reaction. The resulting entry clone was Sanger-sequenced for validation.

The DNA fragments of the highly conserved region encompassing rs55705857, with the risk and non-risk alleles were amplified from genomic DNA extracted from a risk and non-risk carrier’s blood sample, using primers GWL_R55FREV (GGTACCGAGCTCTTACGCGTGCTAGCCCATCAACTGGATTGTGTGCTGGTCAGGATGA) and GWL_R55FREV (AGAGAAATGTTCTGGCACCTGCACTTGCACTGGGGACAGCCTTATGTGAGCAGTATTGCAGTCACTA), which included at their 5′ the B1 and B2 Gateway cloning recombination sequences, respectively. The product was PCR-purified, using QIAquick PCR Purification Kit, and was inserted via PCR cloning upstream of the Gateway (GW) cassette, into the pGW-Luc destination vector. The final vectors were Sanger-sequenced for validation. The MYC promoter in the entry clone was then recombined into the destination vector pGW-Luc, with the highly-conserved fragments inserted upstream of the recombination site, via Gateway LR reaction.

The resulting expression vectors (approximately 600 ng/well), together with a Renilla reporter pRL-CMV (0.6 ng/well), were then used for transfection of HEK293 cells, using Lipofectamine 2000 Transfection Reagent (Invitrogen, Carlsbad CA). The transfections were performed in triplets for each vector (including a triplet of a vector containing no promoter at all, as a negative control) in a 96-well plate. After approximately 48–72 hours following transfection, luciferase expression was measured via the Dual-Glo Luciferase Assay System (Promega, Madison, WI).

### Study approval

The study was approved by Acıbadem University’s institutional review board (ATADEK). Written consent was received from the patients prior to their inclusion in the study. All methods were carried out in accordance with the approved guidelines.

## Additional Information

**How to cite this article**: Oktay, Y. *et al*. IDH-mutant glioma specific association of rs55705857 located at 8q24.21 involves MYC deregulation. *Sci. Rep.* **6**, 27569; doi: 10.1038/srep27569 (2016).

## Supplementary Material

Supplementary Information

Supplementary Table 1

Supplementary Table 2

Supplementary Table 3

Supplementary Table 4

Supplementary Table 5

Supplementary Table 6

Supplementary Table 7

## Figures and Tables

**Table 1:** rs55705857 is not associated with generalized cancer susceptibility.

Cancer	n	MAF(%)	OR (95% CI)	p-value
Colorectal	46	1.09	0.60 (0.07 – 4.78)	0.64
Breast	69	1.45	0.83 (0.18 – 3.82)	0.81
Lung	55	2.73	1.60 (0.43 – 5.93)	0.48
Prostate	32	3.13	1.85 (0.39 – 8.73)	0.43
Haematologic	143	0.35	0.19 (0.03 – 1.53)	0.12
Other CNS tumors	31	0.00	–	–
Other systemic cancers	35	2.85	1.68 (0.36 – 7.91)	0.51
All non-glial malignancies	411	1.34	0.74 (0.33 – 1.78)	0.47

109/143 patients with haematologic malignancies are acute myeloid leukemia (AML) patients.

MAF: minor allele frequency; OR: odds ratio; n: number of patients; CNS: central nervous system.

**Table 2:** rs55705857 is not associated with other IDH-mutant cancers.

Cancer	n	MAF(%)	OR (95% CI)	p-value		
CHS	73	6.14	0.99 (0.39–2.56)	0.99		
LGCT	47	2.13	0.32 (0.07–1.49)	0.14		
British Population*	89	6.59	–	–		
	**Ollier**	**Maffucci**	**Coeliac**	**Solitary**	**TOTAL**	**British Control**
A/G	0	0	0	11	11	11
A/A	17	2	1	89	109	78
*MAF (%)*	*0*	*0*	*0*	*0*	*4.80*	*6.59*
*Odds ratio*					*0.72*	
*p-value*					*0.46*	

***Upper panel**:* IDH-mutant tumor patients grouped according to type of tumor.

***Lower panel**:* IDH-mutant tumor patients grouped according to presence of Ollier, Maffucci or Coeliac syndromes.

^*^British population data was obtained from 1000 Genomes Project Phase I.

*CHS: chondrosarcoma; LGCT: low-grade cartilaginous tumor; MAF: minor allele frequency; OR: odds ratio; n: number of patients.*
