# Supplementary material for: IDH-mutant glioma specific association of rs55705857 located at 8q24.21 involves MYC deregulation
Source: Sci Rep. 2016 Jun 10;6:27569. doi: 10.1038/srep27569 (PMC4901315; doi:10.1038/srep27569)

SUPPLEMENTARY INFORMATION

IDH-mutant glioma specific association of rs55705857 located at 8q24.21 involves MYC deregulation

Yavuz Oktay, Ege Ülgen, Özge Can, Cemaliye B. Akyerli, Şirin Yüksel, Yiğit Erdemgil, İ. Melis Durası, Octavian Ioan Henegariu, Paolo Nanni, Nathalie Selevsek, Jonas Grossmann, Zeynep Erson-Omay, Hanwen Bai, Manu Gupta, William Lee, Şevin Turcan, Aysel Özpınar, Jason T. Huse, M. Aydın Sav, Adrienne Flanagan, Murat Günel, O. Uğur Sezerman, M. Cengiz Yakıcıer, M. Necmettin Pamir, Koray Özduman

Supplementary Materials & Methods

Genotyping of patients and controls

Point mutations were detected by PCR and Sanger sequencing of target region or by "mini-sequencing" using SNaPshot Multiplex System on ABI PRISM 3100 (Applied Biosystems, ThermoFisher, Carlsbad, CA). Rs55705857 genotype was determined by Sanger, mini-sequencing and/or restriction fragment-length polymorphism (RFLP) method. G-allele creates PstI recognition site CTGCA**G**, whereas A-allele creates CTGCA**A** sequence. 304 bp region encompassing rs55705857 was amplified by PCR using primers rs55705857-2F (5'- AGTTGGCTTTGTGGGTTGCT -3') and rs55705857-2R (5'- TCTTTCTCCCAGGGGCTGTA -3'). Recombinant Taq DNA polymerase (MBI Fermentas, Vilnius, Lithuania) and its standard buffer were used in PCR reaction with addition of dNTPs, $MgCl_2$, gDNA at the recommended concentrations. PCR conditions were as follows: 95 °C 2:00, 94 °C 0:30, 59 °C 0:45, 72 °C 0:30, 36 cycles, 72 °C 5:00, 4 °C hold. PCR product was incubated with PstI-HF (New England Biolabs, NEB) enzyme for >2hr and the bands were visualized on 2 % agarose gel. Presence of 177 bp band indicated G-allele. Validity of RFLP was verified by sequencing of 100 RFLP-tested samples (100% concordance).

All samples underwent routine IDH testing by IHC (anti-IDH1-R132H) and by molecular methods (mini-sequencing and/or Sanger sequencing). TERTp-C228T and TERTp-C250T mutations were assessed by mini-sequencing.

Mini-sequencing primers used for rs55705857 and IDH1-R132 are as follows: rs55705857 PCR1F (5'- CAATGCCAGGAGCTTACAAAGAC -3'), rs55705857 PCR1R (5'- CTTGTAAGCTGTGTTGAATGAGATTCT -3'), rs55705857 MSQR (5'- TGGCCCTTTGTTCAAGRAATGCAT); IDH1 EXON4 1F (5'- ACCAAGGATGCTGCAGAAGCTAT -3'), IDH1 EXON4 2R (5'- TACCTTGCTTAATGGGTGTAGATACCA -3'), IDH1 R132G,S,C F (5'- TGGGTAAAACCTATCATCATAGGT -3'), IDH1 R132L,H,P R (5'- TTTTATGACTTACTTGATCCCCATAAGCATGA -3'). IDH2-R140 and IDH2-R172 mutations were assessed by Mini-sequencing after nested-PCR amplification. First round primers used were: IDH2 EXON4 1F (5'- CTGTCCTCACAGAGTTCAAGCTGAAG -3') and IDH2 EXON4 2R (5'- CAGGTCAGTGGATCCCCTCTCCA -3'). Second round primers were: IDH2 EXON4 3F (5'- AGATGTGGAAAAGTCCCAATGGAACT -3') and IDH2 EXON4 4R (5'- TCCACCCTGGCCTACCTGGTC -3'). IDH2 Miniseq primers were: IDH2 R140Q,L G>A,T (5'- TGGAAAAGTCCCAATGGAACTATCC -3'), IDH2 R140W C>T (5'-

TTTTTGTGGAAAAGTCCCAATGGAACTATC -3’), IDH2 R172K,M G>A,T (5’-TTTTTCCCTGGCTGGACCAAGCCCATCACCATTGGCA -3’), IDH2 R172W A>T (5’-TTTTTTTTTTTTCCCTGGCTGGACCAAGCCCATCACCATTGGC -3’).

TERTp-C228T and TERTp-C250T mutations were assessed by mini-sequencing using the following primers: *TERT* PRMT 3F (5’- GCGGAAAGGAAGGGGAGGGGCT -3’), *TERT* PRMT 4R (5’-CTTCACCTTCCAGCTCCGCCTCCT -3’), TERTp-C228T (5’- GAGGGGCTGGGAGGGCCCGGA -3’), TERTp-C250T (5’- CGCGGACCCCGCCCCGTCCCGACCCCT -3’).

Quantitative real-time PCR (qPCR) with TaqMan assays

Total RNA was isolated from tumor samples stored in RNA-later or liquid nitrogen. Tumor samples were homogenized with the help of QIAshredder homogenizer (QIAGEN) and total RNA was isolated with RNeasy Mini kit (QIAGEN). RNA-quality and quantity was determined with NanoPhotometer™ Pearl (Implen GmbH, Munich, Germany) UV-vis spectrophotometer. cDNA was synthesized from 350 ng total RNA in a 20 µl *reaction* by using random primers and HighCapacity cDNA Reverse Transcription Kit (Applied Biosystems, ThermoFisher, Carlsbad, CA). 35-53 ng cDNA was used for quantification of *CCDC26* expression by TaqMan GeneExpression Assay Hs00540885_m1 and normalized to ACTB (beta-actin) assay Hs01060665_g1 with □□Ct method. Both assays span exons. Reaction was carried out using Universal Master Mix and run on ABI7500-FAST Real-Time PCR System (Applied Biosystems, ThermoFisher, Carlsbad, CA) for 42 cycles.

OncoScan analysis

DNA were extracted from FFPE sections according to manufacturer’s instructions using QIAmp DNA mini kit (QIAGEN). Following quality control of DNA samples, standard protocols for OncoScan FFPE Express 2.0 (Affymetrix) were applied and the data was analysed using “Nexus Express for OncoScan” software.

LC-MS/MS analysis

Peptide samples were analyzed by LC-MS/MS using an LTQ-Orbitrap Velos hybrid mass spectrometer (Thermo Fischer Scientific, Bremen Germany) coupled to an Eksigent-Nano-HPLC system (Eksigent Technologies, Dublin, CA, USA). Peptide were loaded on a self-made frit-column (75 µm × 150 mm) packed with Magic RP C18 AQ, 200A, 3 µm beads (Bischoff GmbH, Leonberg, Germany), coupled to a fused-silica emitter (20 µm x 8 cm, Tip: 10±1 µm; New Objective, Woburn, MA, USA). Solvent composition was 0.1% formic acid for channel A, and 0.1% formic acid and 99.9% acetonitrile for channel B. Peptides were eluted at a flow rate of 300nL/min by a gradient of 2 to 35% ACN in 125 min, 35-40% in 5 min, 40-96% in 4 minutes. Full-scan MS spectra (300−1700 m/z) were acquired at a resolution of 60000 at 400 m/z after accumulation to a target value of 1e6. Collision-induced dissociation (CID) fragmentation was performed on the twenty most intense signals per cycle. CID spectra were acquired using a normalized collision energy of

35 and a maximum injection time of 100 ms. Charge state screening was enabled and singly and unassigned charge states were rejected. Precursor masses previously selected for MS/MS were excluded from further selection for 60 s, and the exclusion window was set to 10 ppm. The size of the exclusion list was set to a maximum of 500 entries. The samples were acquired using internal lock mass calibration on m/z 429.088735 and 445.120025. A pool containing 0.5 µl of each sample was analyzed and used as reference for the following data analysis.

Protein identification and protein quantification using Progenesis

The raw files from the mass spectrometer were loaded into ProgenesisLCMS (v.4.0.4265.42984). Before the automatic aligning, the loading and the wash phase of the gradient were cut, the aligning reference was chosen as such where the most features could visually be seen. From each ProgenesisFeature (default sensitivity in peak picking) a maximum of the top five tandem mass spectra were exported using charge deconvolution and deisotoping option and a maximum number of 200 peaks per MS/MS. The Mascot generic file (.mgf) was searched with Mascot Server v.2.4.1 (www.matrixscience.com) using the parameters 10ppm for precursor ion mass tolerance, 0.6 Da for fragment ion tolerance. Trypsin was used as the protein-cleaving enzyme, two missed cleavages were allowed. Carbamidomethylation of cysteine was specified as a fixed modification, and oxidation of methionine, pyroglutamate formation from glutamine, deamidation from glutamine and asparagine and N-terminal acetylation of proteins were selected as variable modifications.

Searched was a forward and reversed human database (downloaded on 18/04/2013 from uniprot) concatenated to 260 known mass spectrometry contaminants in order to evaluate the false discovery rate using the target-decoy strategy (1).

The mascot result was loaded into Scaffold v4.1.1 using 95% PeptideProphet and ProteinProphet thresholds and protein cluster analysis. The spectrum report was exported and loaded into ProgenesisLCMS.

In the experimental design, we used a between group analysis where two following experimental designs were generated: tumor samples from 9 IDH-mutant 1p/19q co-deleted ATRX-wt WHO grade-II DGs (n=6 vs. n= 3, A/A and A/G groups, respectively) and 7 IDH-mutant 1p/19q-intact ATRX-mut WHO grade-II DGs (n=4 vs. n= 3, A/A and A/G groups, respectively) were used for proteomic analysis. Normalization was kept with default settings. For quantification, we assessed all proteins identified with at least 2 features. Proteins were grouped with Progenesis and only non-conflicting features were used for quantification.

Transcriptional regulation networks that were differentially regulated were identified by employing “transcription regulation” function of GeneGo MetaCore version 6.21 build 66768 from Thomson Reuters, which centers created sub-networks on transcription factors. Z-score and g-scores (>100) were used to identify transcription factors with highest difference between A/G and A/A groups of tumors.

RNA-seq analysis

Total RNA was extracted in the same way as for qPCR and analyzed by using RNA 6000 Nano Kit on Bioanalyzer 2100 (Agilent) before being transported on dry-ice to BGI-Tech (Shenzhen, China) overnight. Library construction, sequencing and preprocessing of raw data were performed by BGI-tech. The preprocessing of raw data included the following: 1. Removing reads with adaptor sequences. 2. Removing reads in which the percentage of unknown bases (N) is greater than 10%. 3. Removing low quality reads. (If the percentage of the low quality base (base with quality value ≤ 5) is greater than 50% in a read, it's defined as low quality).

The filtered single-end 50bp-long reads from libraries from the seven tumor tissues were then processed together. The reads were mapped against the hg19 human genome using TopHat (version 2.0.11) (2,3), using only known exon-exon junctions provided by Illumina iGenomes with all the other parameters set to their default values. Quality control of alignments was performed using RNA-SeQC (version 1.1.7). The TopHat aligned reads were then assembled individually using Cufflinks (version 2.2.1) (2), using the Illumina iGenomes reference annotation, i.e. not assembling novel assemblies. After the final transcriptome assembly using Cuffmerge, expression levels were quantified using Cuffquant (version 2.2.1), providing the hg19 multifasta file, hence using the bias detection and correction algorithm and using the “multi-read-correct” option to more accurately weight reads mapping to multiple locations in the genome Finally, differential expression analysis was performed using Cuffdiff (version 2.2.1). The differential expression results were then analyzed using CummeRbund (v2.7.2) (2).

1. Käll L, Storey JD, MacCoss MJ, Noble WS. Assigning significance to peptides identified by tandem mass spectrometry using decoy databases. J Proteome Res.**7**, 29–3 (2008).
2. Trapnell C, Roberts A, Goff L, Pertea G, Kim D, Kelley DR, et al. Differential gene and transcript expression analysis of RNA-seq experiments with TopHat and Cufflinks. Nat Protoc.**7**, 562–78 (2012).
3. Kim D, Pertea G, Trapnell C, Pimentel H, Kelley R, Salzberg SL. TopHat2: accurate alignment of transcriptomes in the presence of insertions, deletions and gene fusions. Genome Biol.**14**, R36 (2013).

Legends for Supplementary Figures and Tables.

Suppl. Figure 1. Clinicopathologic attributes of WHO grade-II tumors suggest minimal or no difference between rs55705857-G allele carriers compared to non-carriers. (**a**) Comparison of patient age between rs55705857-G allele carriers to non-carriers. WHO grade-II gliomas were classified based on histology or molecular alterations. Classical histology-based classification system was used to identify a tumor's "histopathologic" subtype, while IDH-mutation, ATRX-mutation and 1p19q-codeletion status with histologic grade were used to identify a tumor's "molecular" subtype. (**b**) Comparison of Ki-67 indices between rs55705857-G allele carriers to non-carriers. The same classification strategy as described above was used. Error bars indicate first quartiles and the median. (**c-d**) Kaplan-Meier curves that depict time-to-malignant degeneration (**c**) and overall survival (**d**) for A/G and A/A group of IDH-mutant grade-II oligodendrogliomas and astrocytomas. Log-rank test was used to calculate p-values.

Suppl. Figure 2. Copy number analysis of WHO grade-II IDH-mutant 1p19q-codeleted ATRX-wt diffuse gliomas with by Oncoscan detects no significant difference between tumors with A/G and A/A genotypes.

Suppl. Figure 3. Percentage of IDH-mut WHO Grade-II glioma samples (**a**) positively stained by MYC-IHC, (**b**) with *MYC* copy number gain and (**c**) oncogenic copy number alteration in either *MYC, MAX* or *FBXW7* genes.

Suppl. Table 1: Demographic distribution of glioma patients, other cancer patients and controls.

Suppl. Table 2. Rs55705857 genotype distribution by glioma subtype.

Suppl. Table 3. OncoScan analysis results of 22 WHO grade-II *IDH*-mutant oligodendrogliomas, A/A (n=18) and A/G (n=4) genotypes.

Suppl. Table 4. List of genes differentially expressed between A/G and A/A groups of WHO grade-II IDH-mutant 1p19q-codeleted ATRX-wt diffuse gliomas analyzed by RNA-seq.

Suppl. Table 5. *PANOGA* based network analysis of RNA-seq data suggests differential regulation of neurotrophin and Ras/MAPK pathways between A/G and A/A groups of WHO grade-II IDH-mutant 1p19q-codeleted ATRX-wt gliomas.

Suppl. Table 6. List of proteins differentially expressed between A/G and A/A groups of WHO grade-II IDH-mutant 1p19q-codeleted ATRX-wt diffuse gliomas as analyzed by LC-MS/MS, cut-off p-value is 0.05 (ANOVA).

Suppl. Table 7. List of proteins differentially expressed between A/G and A/A groups of WHO grade-II IDH-mutant 1p19q-intact ATRX-mut diffuse gliomas as analyzed by LC-MS/MS, cut-off p-value is 0.05 (ANOVA).

Supplementary Figure 1.

(glioma-MYC) *Oktay et al.*

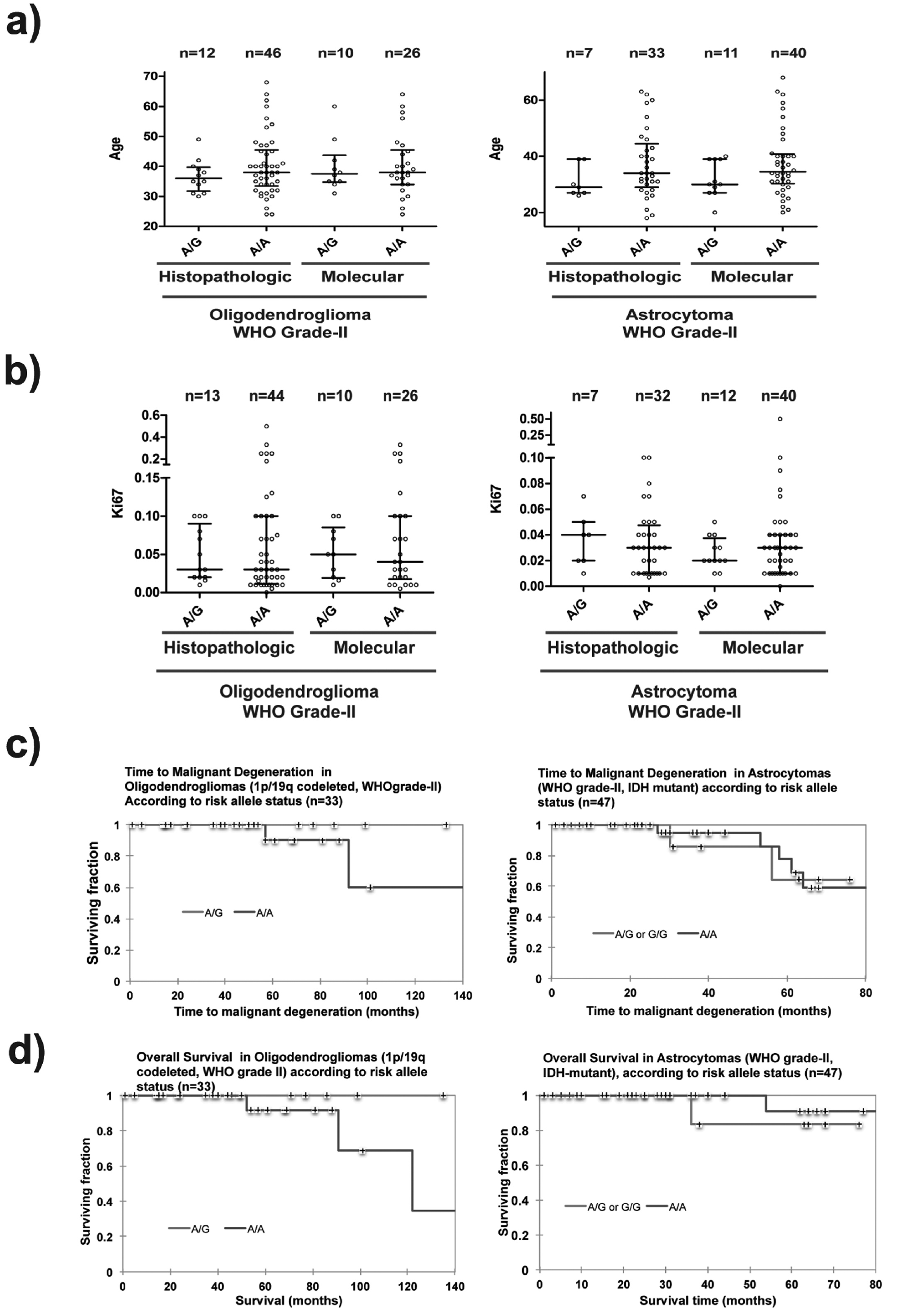

Supplementary Figure 2.

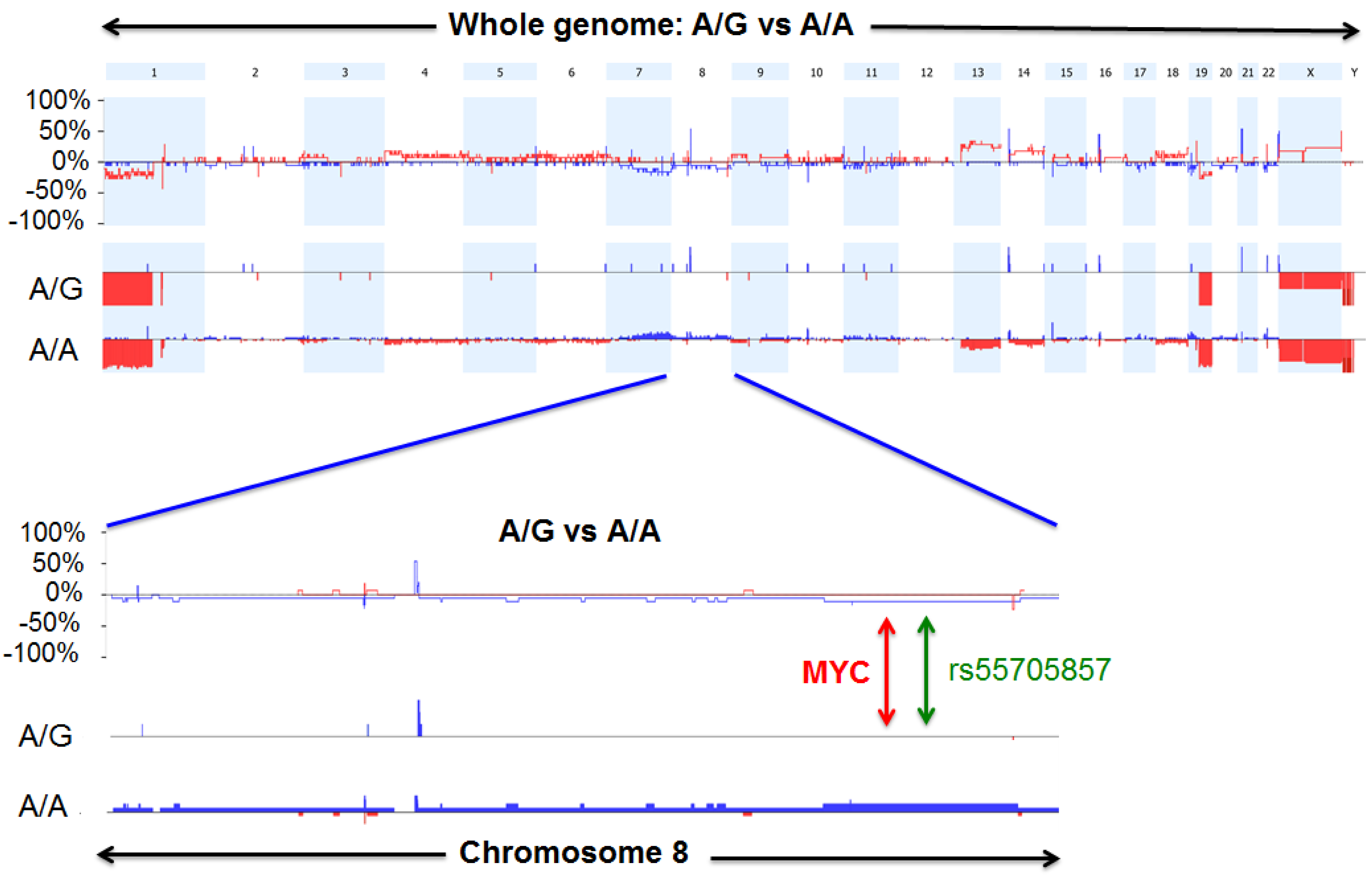

Supplementary Figure 3.

a)

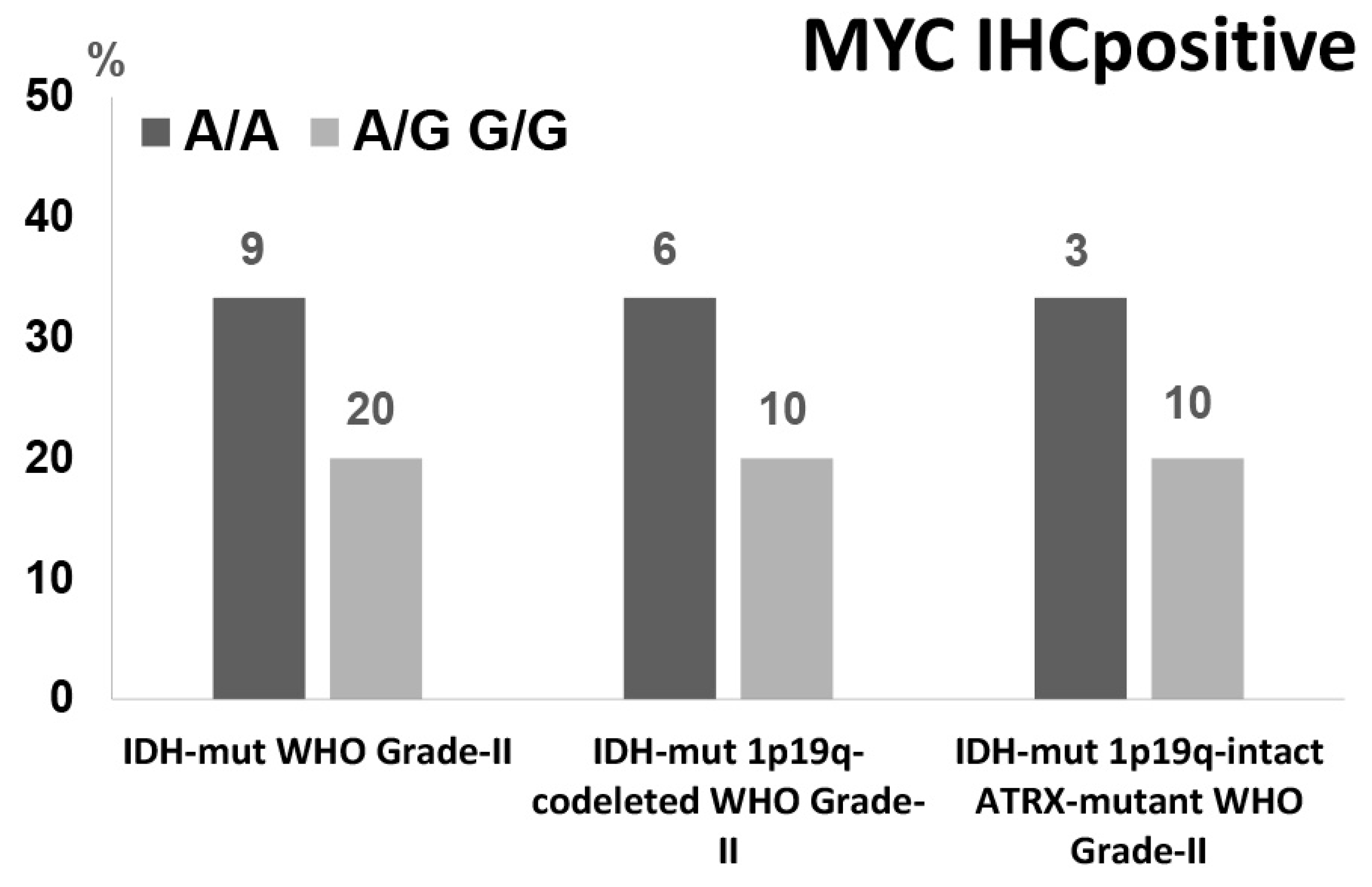

b)

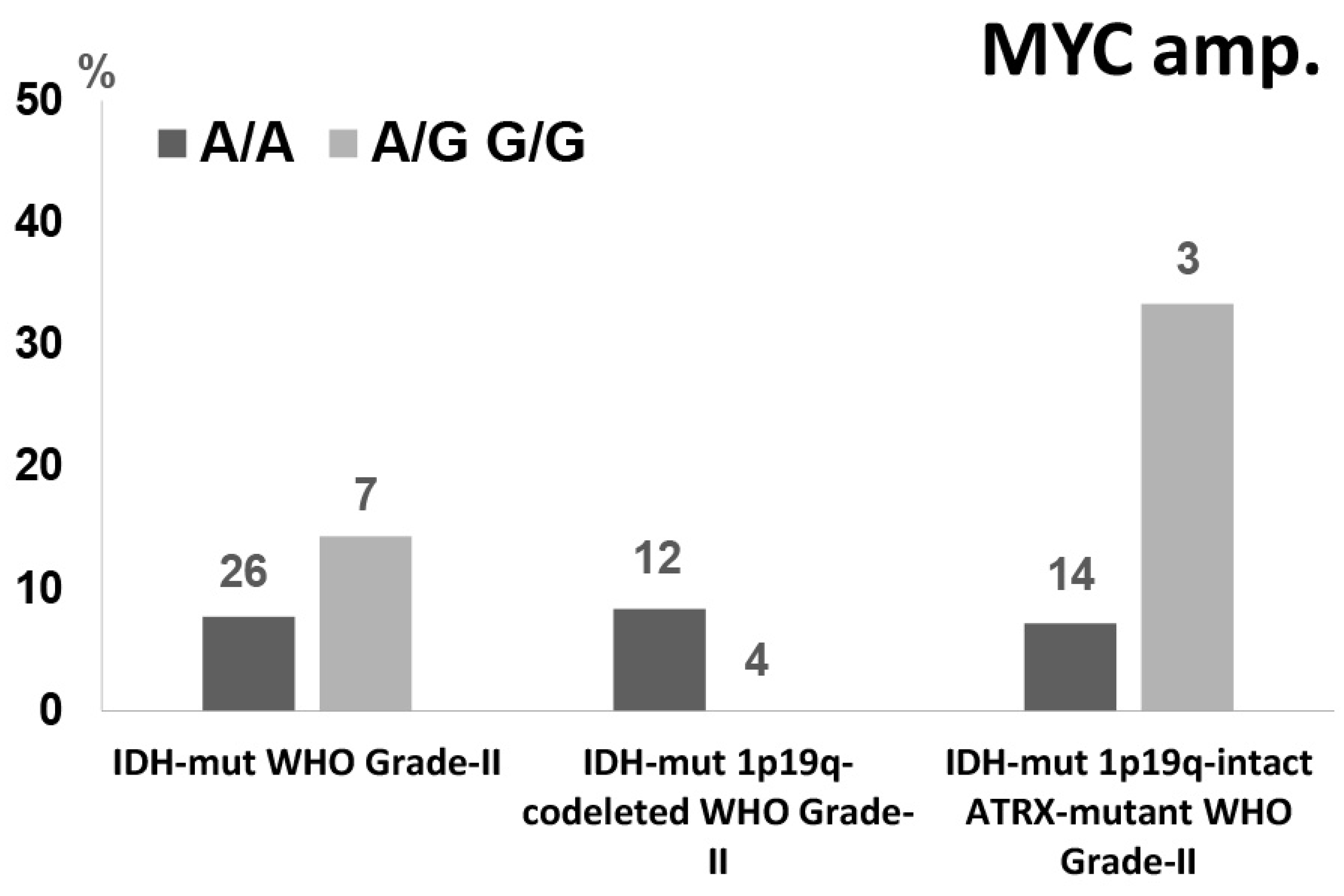

c)

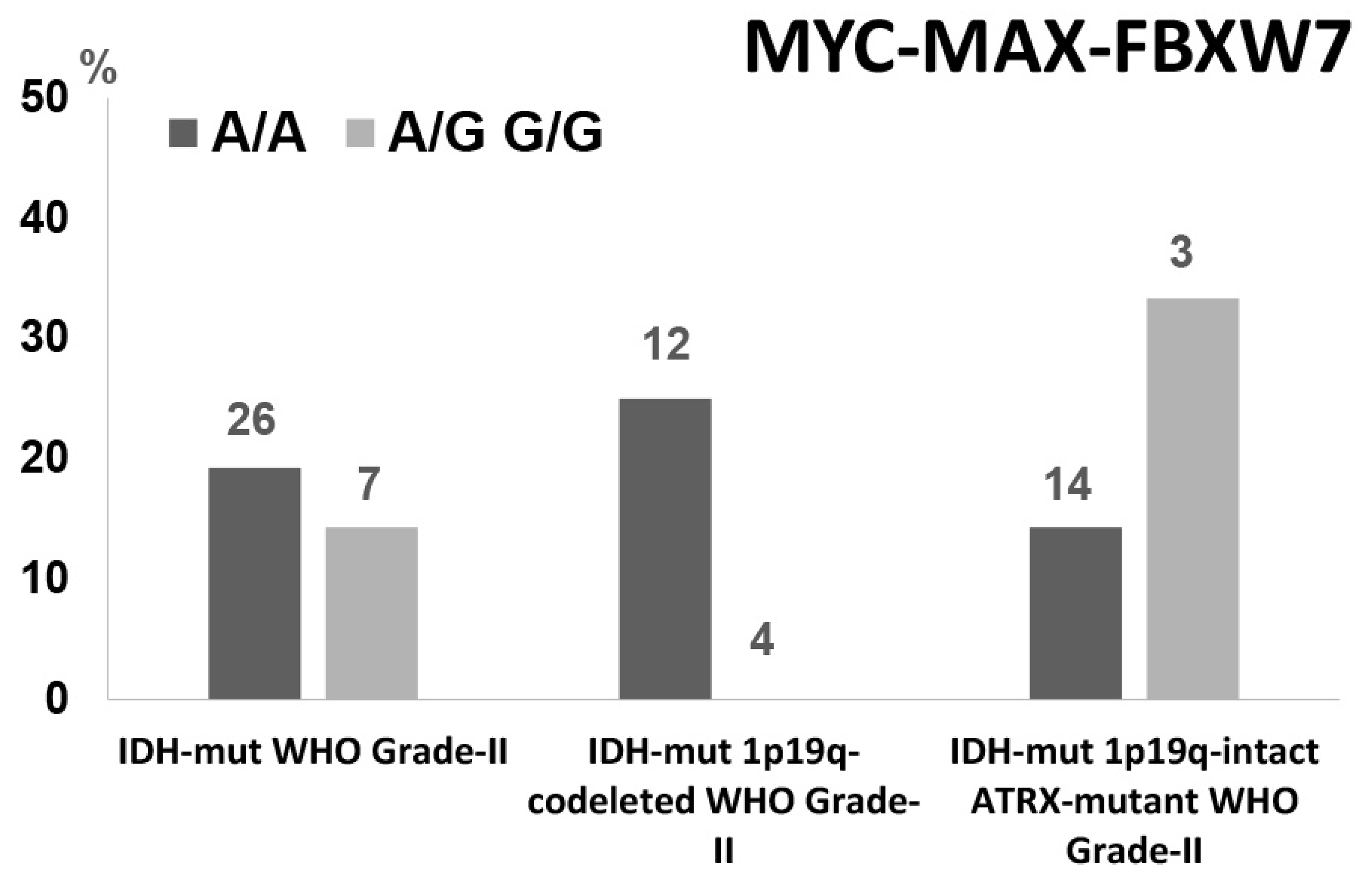

Supplement: Supplementary Information [file srep27569-s1.pdf]
